# Influence of goat management systems on hematological, oxidative stress profiles, and parasitic gastrointestinal infection

**DOI:** 10.14202/vetworld.2023.483-490

**Published:** 2023-03-17

**Authors:** Charinya So-In, Nuchsupha Sunthamala

**Affiliations:** 1 Department of Veterinary Technology, Faculty of Agricultural Technology, Kalasin University, Kalasin 46000, Thailand; 2 Department of Biology, Faculty of Science, Mahasarakham University, Mahasarakham, 44150, Thailand

**Keywords:** goat, hematology values, management systems, oxidative stress, parasites

## Abstract

**Background and Aim:**

Good management in goats is known for good quality health and increasing productivity. The physiological change studies in goats are limited despite some existing studies on the relationship of various patterns to growth rates. This study aimed to determine the hematological parameters, oxidative stress, and parasitic infection in three management systems in Thai native goats.

**Materials and Methods:**

A total of 18 male goats were randomly assigned to the three systems: The free-range model (FREE), the semi-intensive model (SEMI), and the kept-in-a-cage model (BARN) for 35 days. Blood, fecal sampling, and weight data were collected and monitored every 5 days for analysis.

**Results:**

No statistical differences were found in the FREE and SEMI groups, but significance was observed in the BARN group. The body weight of the goats gradually reduced from 13.0 ± 2.44 kg to 10.18 ± 2.61 kg (mean ± standard deviation). In contrast, the significantly increasing red blood cells, packed-cell volume, white blood cells, neutrophil-to-lymphocyte (N/L) ratio, cortisol hormone, and antioxidation profiles were observed to be higher in BARN than in FREE and SEMI groups. The intensity of the parasite eggs was remarkably significant. It was observed in the BARN group between the beginning and end of the experiment (supported by a high level of eosinophils).

**Conclusion:**

These data can be applied for the realistic evaluation and improvement of management practices, especially fully restrained management (BARN) for monitoring the health status of goats.

## Introduction

Nowadays, goat production has become a significant contributor to the agricultural economy of Thailand, which continues to grow and has been recorded as 1 million heads in 2019, predominantly comprising meat-producing animals [1]. Tethering, free range, semi-free range, intensive, semi-rotational grazing, and livestock-tree crop integration are the traditional goat management systems in Thailand [2]. The free-range management system is the most popular system typically practiced by small farm holders (<60 animals per herd). It allows goats to browse alongside road verges, empty paddy fields, ample uncultivated areas, and use public lands [3]. With the semi-free-range system, the goat forage in the grazing area, but the herd returns to the shelter in the evening and is kept in small hutches overnight for protection [4]. In the intensive system, goats are continuously confined to housing with limited access to land. Farmers are shifting to intensive management systems to meet the increasing demand for goat production, which involves the total confinement of animals, resulting in the restriction of movement and other natural behaviors of animals [5, 6]. These production systems are likely to impose various stress levels on goats, and imbalance between antioxidants, and free radicals within cells, resulting in oxidative damage to proteins, lipids, and tissues from multiple organs [5, 7, 8].

The optimal level of reactive oxygen species in an organism is controlled by the cellular antioxidant protection system comprising enzymatic and non-enzymatic elements. When the system is insufficient, the organism is subjected to increasing levels of oxidative stress, resulting in a cascade of pathological processes with highly negative consequences [9, 10]. Furthermore, long-term stress can lead to chronic stress, which causes a decrease in growth rate and more aggressive behavior, affecting internal organs such as the liver, which are sensitive to enzymatic changes. The effects of oxidative stress can be studied by measuring lipid peroxidation, blood value changes, and cortisol hormone levels [10, 11, 12]. The elevation of glucocorticoids activated from the hypothalamus−pituitary−adrenal axis is one of the primary physiological mechanisms of vertebrates that cope with challenging environmental stressors, which reduces the immune response efficiency and can lead to a high intensity of parasitic infection [12].

This study aimed to investigate the effects of goat farming patterns (goat management) on hematological parameters, parasitic infection, and oxidative stress. There are currently no physiological studies of changes in goats, despite some existing studies on the relationship of different cultural patterns to growth rates. The findings of this study can potentially help to develop and improve the efficiency of management methods in the future.

## Materials and Methods

### Ethical approval

The study was performed under the supervision of a veterinarian and in accordance with a proposal approved by Kalasin University’s Committee on Ethics and Standards for Raising and Using Animals for Scientific Purposes (KSU13/2559).

### Study period and location

The study was conducted from November to December 2019 (winter season) at the Institute of Veterinary Technology, Agricultural Technology, Kalasin University.

### Experimental animals

The study was conducted at the Institute of Veterinary Technology, Agricultural Technology, Kalasin University. Eighteen male Thai native goats aged 3 months, all from the farm at Kalasin University, with similar body weight (13.18 ± 2.23 kg; mean ± standard deviation) were divided into three groups (six goats per group): (1) The group raised by natural release or the free-range model, which allowed the goats to feed themselves naturally (FREE), (2) the group with the semi-intensive model, which was raised in houses since 6 pm–6:59 am and on the ground between 7 am and 5:59 pm with a standard diet (SEMI), and (3) the group kept in a cage (1 goat/m^2^), which was raised within a barn all the time by providing water and food inside the stall which had a raised and sloped stable floor to maintain health (BARN) [3–5].

The goats were given a 5-days rest period before the experiment. Blood collection and fecal sampling (triplicate samples from each period) were performed and analyzed to detect parasitic infections. The weighing process included growth monitoring every 5 days at 7:00 am. All groups of goats received ad libitum feeding (18% crude protein) and water at an average temperature of 25°C and approximately 12 h of daily natural light throughout the winter season in Thailand. Blood samples were drawn from the jugular vein and transferred to iced tubes containing the anticoagulant agent ethylenediaminetetraacetic acid (EDTA). Plasma was obtained from the blood by centrifugation at 400× *g* for 10 min at 4°C and was stored at −20°C for further analysis at the Hematology Laboratory, Faculty of Veterinary Medicine, Khon Kaen University, Thailand.

### Determination of antioxidant enzyme activity

Antioxidant enzyme activities were determined using colorimetric methods. Briefly, chloroform and ethanol were used to remove the supernatant from the blood samples. The supernatant was mixed with xanthine, EDTA, Na_2_CO_3_, nitrotetrazolium blue chloride, and bovine serum albumin. Xanthine oxidase was added as required. The same procedure was used for comparison with the bovine CuZn-superoxide dismutase (SOD) standard. CuCl_2_ was used to stop each reaction after 20 min of incubation at 25°C. A wavelength of 550 nm was used in the formazan absorbance test. Comparative metrics between the supernatant and the SOD standard were based on the degree of formazan inhibition. The level of SOD activity was determined by the degree of inhibition of formazan formation [13, 14].

Catalase (CAT) activity was measured as follows. The blood sample was incubated in a hydrogen peroxide (H_2_O_2_) substrate for 1 min at 37°C before the reaction was stopped with ammonium molybdate, according to the method given by the homogenate. The yellow complex was measured at a wavelength of 405 nm and its intensity was compared to the CAT standard [13, 14].

The measurement of the activity of glutathione peroxidase (GPx) using sample homogenate, 0.02 mM EDTA, 8.26 mM, sodium azide, and 2.48 mM sodium phosphate buffer (pH 7.4) was mixed and incubated for 10 min at 30°C. Glutathione (GSH) was then applied at a concentration of 1.24 mM H_2_O_2_ 3.31% (w/v) Sulfosalicylic Acid (SSA) that was used to initiate the reaction, which was stopped by adding SSA. The reaction mixture was centrifuged at 1500× *g* for 15 min. The activity of GPx was measured using blood supernatant [13, 14].

### Measurement of GSH content

The blood samples were deproteinized with SSA and centrifuged at 10,000 g at 4°C for 10 min after being held at 2°C–8°C for 10 min. The reaction mixture (EDTA, potassium phosphate buffer (pH 7.0)), GSH reductase, 5,5’-dithiobis-(2-nitrobenzoic acid), and nicotinamide adenine dinucleotide phosphate were mixed with the supernatants to determine the total GSH. A spectrophotometer measured the absorbance of the thiol anions at a wavelength of 405 nm every 60 s for 5 min.

The total GSH content was calculated by comparing the A405/min (slope) of the sample to that of the GSH standard. The GSH contents were calculated by subtracting the total GSH contents from the Glutathione disulfide (GSSG) — a comparison of 4-vinyl pyridine incubation with an aliquot of the sample supernatant and GSSG standard incubation at room temperature (25°C) for 60 min. The procedure for determining GSSG content was similar to that for determining GSH content [14, 15].

### Determination of lipid peroxidation

A thiobarbituric acid (TBA) assay was used to assess lipid peroxidation. The reaction mixture (trichloroacetic acid, acetic acid, and 2-TBA) was added after the blood sample. The malondialdehyde (MDA) standard was incubated for 1 h at 37°C and the samples were boiled for 15 min. The TBA reactive species (or TBARS) were measured using a spectrofluorometer with an emission wavelength of 551 nm and an excitation wavelength of 528 nm [15].

### Parasitological analyses

One gram of fresh fecal samples (collected directly from the rectum) from formalin-preserved samples was concentrated through fecal sedimentation to assess gastrointestinal parasites. The entirety of the sediment was analytically examined at 10× objective light magnification, and the helminth eggs were counted as eggs per gram in triplicate samples for each goat [16].

### Statistical analysis

The experimental results were analyzed using two-way analyses of variance according to Tukey’s multiple comparison tests (Prism version 9, GraphPad Soft Inc. La Jolla, CA, USA). p ≤ 0.05 was considered statistically significant.

## Results

### BARN raising goats reduce body weight

**Figure-1:**
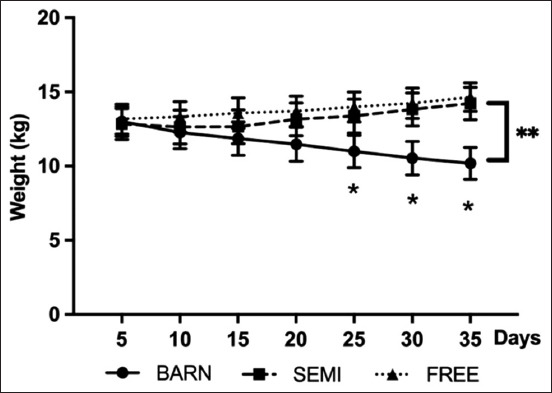
Body weight of goats under three management systems (BARN, SEMI, and FREE). The data are presented as the mean ± standard deviation based on six goats per system with four replicates in each. Different symbols indicate a significant difference among different weight levels (*p ≤ 0.05, **p ≤ 0.01).

Concerning the influence of the management systems on the weight of goats, there was no statistical difference in the trend of mean goat weight in the FREE and SEMI groups (p > 0.05), but significance (p ≤ 0.01) was observed in the BARN group. Body weight was significantly reduced from 13.0 ± 2.44 kg to 10.18 ± 2.61 kg (Figure-1).

### Hematological parameters and serum biochemical analysis of goat raising in three systems

**Figure-2:**
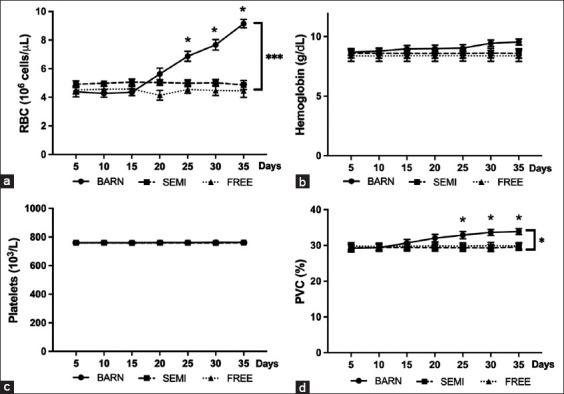
Hematological profiles of goats under three management systems (BARN, SEMI, and FREE). (a) Red blood cell counts, (b) hemoglobin, (c) platelets, and (d) packed-cell volume percentage. The data are presented as the mean ± standard deviation based on six goats per system with four replicates in each. Different symbols indicate a significant difference among different levels (*p ≤ 0.05, **p ≤ 0.01, ***p ≤ 0.001).

**Figure-3:**
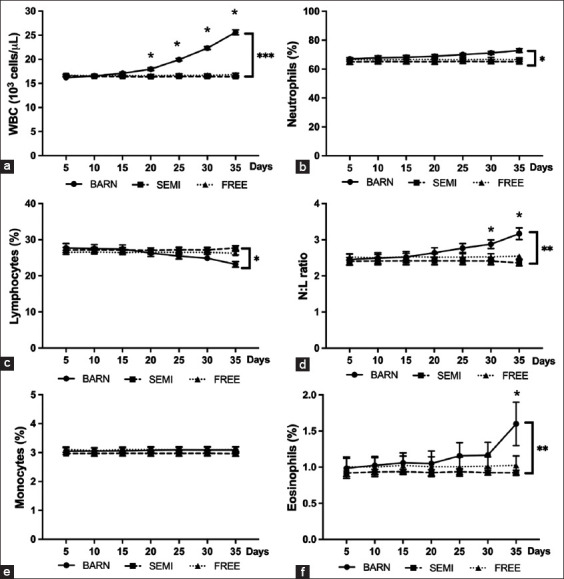
Hematological profiles of goats under three management systems (BARN, SEMI, and FREE). (a) WBCs counts, (b) % neutrophil, (c) % lymphocytes, (d) neutrophil-to-lymphocyte ratio, (e) % monocyte, and (f) % eosinophil. The data are presented as the mean ± standard deviation based on six goats per system with four replicates in each. Different symbols indicate a significant difference among different levels (*p ≤ 0.05, **p ≤ 0.01, ***p ≤ 0.001).

The hematological parameters in the two groups (FREE and SEMI) did not differ significantly (Figures-2b and c). Contrary to Figures-2a (p ≤ 0.001) and 2d (p ≤ 0.05), statistically significant results were observed in the BARN group, especially the increase in red blood cells (RBCs) and packed-cell volume (PCV). The white blood cell (WBC) count profile was significantly higher than FREE and SEMI goats (Figure-3a) (p ≤ 0.001). Similarly, in Figures-3b–d (p ≤ 0.05, p ≤ 0.05, and p ≤ 0.01), only the neutrophil-to-lymphocyte (N/L) ratio of WBCs (corresponding to neutrophil and lymphocytes) in the BARN group was significantly increased (with low values of lymphocyte and high values of neutrophil). Interestingly, it is shown in Figure-3f (p ≤ 0.01) that the percentage of eosinophils was gradually induced in the BARN group. However, the percentage of monocytes was not different in all groups (Figure-3e).

**Figure-4:**
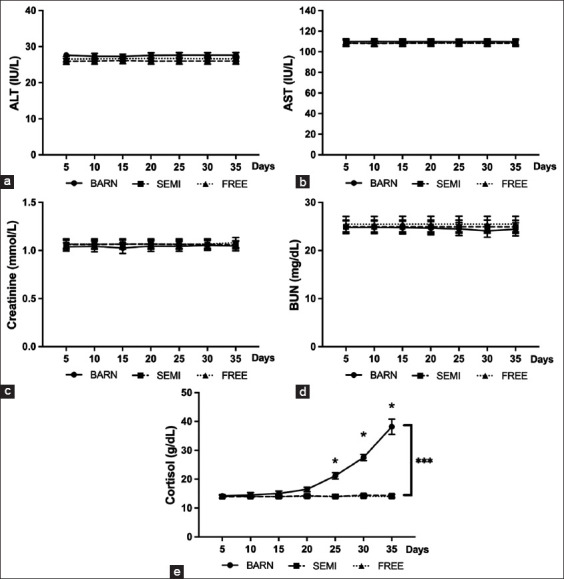
Biochemical profiles of goats under three management systems (BARN, SEMI, and FREE). (a) Alanine aminotransferase, (b) aspartate aminotransferase, (c) blood urea nitrogen, (d) creatinine, and (e) cortisol hormone. The data are presented as the mean ± standard deviation based on six goats per system with four replicates in each. Different symbols indicate a significant difference among different levels (*p ≤ 0.05, ***p ≤ 0.001).

On the other hand, the serum biochemical analysis of housing stressed goats for liver and kidney functions revealed non-significant changes in alanine aminotransferase, aspartate aminotransferase, blood urea nitrogen, and creatinine in the control group (Figure-4a-d). Moreover, a significant increase in cortisol levels was found in the BARN group, as shown in Figure-4e (p ≤ 0.001).

### Antioxidant profiles of goats under the three management systems

**Figure-5:**
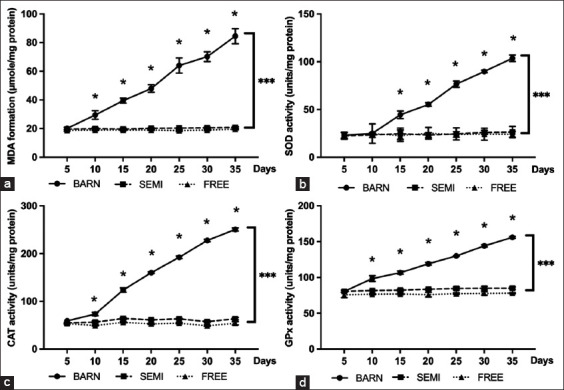
Antioxidant profiles of goats under three management systems (BARN, SEMI, and FREE). (a) Lipid-peroxidation (malondialdehyde), (b) superoxide dismutase activity, (c) catalase activity, and (d) glutathione peroxidase activity. The data are presented as the mean ± standard deviation based on six goats per system with four replicates in each. Different symbols indicate a significant difference among different levels (*p ≤ 0.05, ***p ≤ 0.001).

Figure-5a (p ≤ 0.001) shows the level of lipid peroxidation using MDA, which was used to indicate oxidative stress. There was no statistical difference between the FREE and SEMI groups. In the BARN group, there was a rapid and significant increase in MDA throughout the experiment. Consistent with the study of three metrics of antioxidant enzyme activity: SOD, CAT, and GPx, in the FREE, and SEMI groups, the antioxidant activities were not substantially different, as observed in Figures-5b–d (all with p ≤ 0.001). However, BARN was able to significantly increase the activities, such that the trends of SOD, CAT, and GPx activities were similar to the trend of MDA.

### Stress induces the intensity of egg parasites

**Figure-6:**
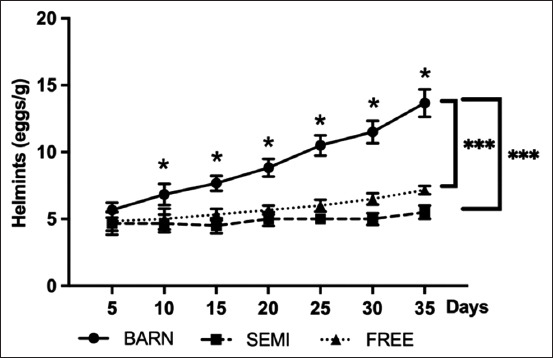
Parasitic eggs count of goats under three management systems (BARN, SEMI, and FREE). The data are presented as the mean ± standard deviation based on six goats per system with four replicates in each. Different symbols indicate a significant difference among different levels (*p ≤ 0.05, ***p ≤ 0.001).

Gastrointestinal nematode eggs were occasionally observed at the beginning of the experiment, indicating an impact of induced stress on the intensity of the egg’s parasite. At the end of the experiment, the FREE group had significantly more nematode eggs than the SEMI group, while a highly significant difference was observed in the BARN group (Figure-6).

## Discussion

The sources of stressors in goats are many, such as cage restraint, hosing management, transportation, seasonality changes, heat, unfamiliar environments, and others [17–19]. After prolonged exposure to stress, goats generate physiological mechanisms that confer them the potential to tolerate stress. Stress causes behavioral and physiological changes and the activation of the adrenomedullary response through the release of catecholamine hormones (epinephrine and norepinephrine). These hormones enhance heart rate, muscle tone, blood pressure, and other physiological and behavioral changes that allow an animal to withstand stressful situations [17]. The second hormonal response is initiated within minutes of the onset of a stressor. A hormonal cascade triggers the synthesis and release of glucocorticoids (cortisol or corticosterone) that have widespread effects on the immune and reproductive systems [17, 20, 21]. The effect of confinement stress on body weight and hematological and biochemical parameters in goats is the target of this study. The goats lost weight gradually in the BARN group, harming the appetite center of the hypothalamus, causing a decrease in feed intake, and entering a stage of negative energy balance. Consequently, the body decreases in weight [20, 22].

Hematological parameters are one of the indicators that can evaluate health status and assess responses to stressful conditions. The significant elevation of RBC counts and %PCV in the BARN group is consistent with the findings of Alam *et al*. [23] and Attia [24]. These authors suggested that the appetite center of the hypothalamus was impacted when the animals were exposed to a high stressor. Consequently, this caused a decrease in feed intake. Subsequently, hemoconcentration developed due to dehydration, which caused erythrocyte release from the spleen and resulted in abnormally higher PCV levels. The increased RBC concentration might have caused the high PCV values [21, 24–26]. During the stressed phase, the number of WBC tended to grow in the BARN group, consistent with research conducted on goats [27] and sheep [28]. However, similar to their reports, the neutrophil counts increased, coupled with decreased lymphocyte counts, in the BARN group of this study, matching with findings from Attia [24] and Adenkola *et al*. [27]. The N/L ratio of goats increased tremendously, like a long-term increase in circulating cortisol, which can be an indication of suffering stress in goats. The extreme secretion of cortisol levels implies stress that results in the elevation of neutrophils caused by shifting the neutrophils to the peripheral circulation from the lymphoid tissues to promote bone marrow formation and synthesize neutrophils [17, 21].

Furthermore, the increase in MDA and antioxidant profile (e.g., SOD, CAT, and GPx) values in blood indicated a stressful condition in goats, especially in the BARN group. This may have been due to limited space, which can increase oxidative damage, resulting in MDA generation, and increased free radical generation and could be indicated in oxidative stress. Antioxidant enzymes are the first line of defense against oxidant reactions. In biological systems, the balance between antioxidants, including but not limited to CAT, SOD, and GPX, and levels of oxidants (such as MDA) are essential to scavenge the oxidative metabolites or to repair the damage caused by oxidative stress in various tissues, including hematopoietic and reproductive organs [28, 29].

When exposure to stressors repeatedly occurs or over a prolonged period, animals may experience a chronic elevation of glucocorticoids, reducing their immune response efficiency and potentially leading to a higher intensity of parasitic infection [30]. There have not been any studies about stressors inducing parasitic intensity. However, the BARN group revealed a significantly increased number of nematode eggs related to stressor exposure. These results are similar to those published by Joó *et al*. [31] in horses, who found that a higher stocking rate was strongly associated with higher egg parasite counts [32]. However, few studies have found a direct relationship between stress and high levels of parasitism [30, 33]. The results confirmed the objective of this study, pointing to the high percentage of eosinophils. Parasitic infections, especially those concerning nematodes that invade tissues, induce a strong Th2-type immune response, which increase immunoglobulin E and eosinophil levels in the blood and tissues [34]. Eosinophils are not effective against all possible helminth infections but are effective against nematode larvae [35].

## Conclusion

This study highlighted the influence of housing systems used for goat management on hematology, stress profiles, and the value of feces egg counts. The study demonstrated that the BARN system was associated with a significantly higher level of three indicators than goats in the FREE and SEMI groups. This result indicates a stressful situation. Thus, this study of goat management on hematology, stress profiles, and parasitism could be used as a model for more efficient management of goat-rearing patterns in the future, in addition to further studies on probable genetic diversity improvement.

## Authors’ Contributions

CS and NS: Conceived and designed the experiments, analyzed the data, contributed reagents/materials/analysis tools, and wrote the paper. CS: Performed the experiments. All authors have read, reviewed, and approved the final manuscript.
